# Transesophageal endoscopic ultrasound-guided aspiration of the left atrium for diagnosis of T-cell lymphoblastic leukemia/lymphoma: a case report

**DOI:** 10.1055/a-2795-7978

**Published:** 2026-02-26

**Authors:** Yao Lu, Duanmin Hu, Jialiang Huang, Tianze Shi, Beinan Hu, Guilian Cheng

**Affiliations:** 1 105860 Department of Gastroenterology, The Second Affiliated Hospital of Soochow University, Suzhou, China

**Fig. 1:**
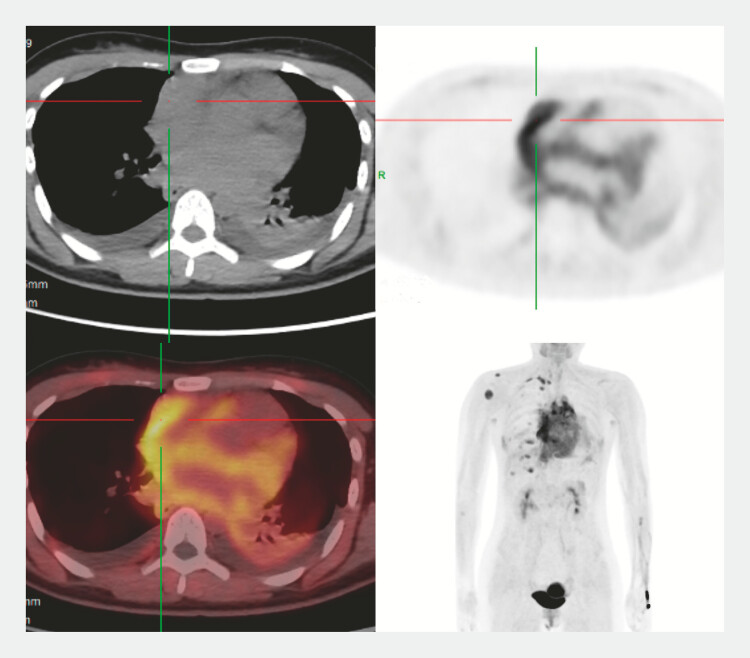
PET/CT showed diffuse metabolic hyperactivity in the heart and local mediastinum, which was considered suspicious for malignancy. PET/CT, positron emission tomography/computed tomography.

**Fig. 2:**
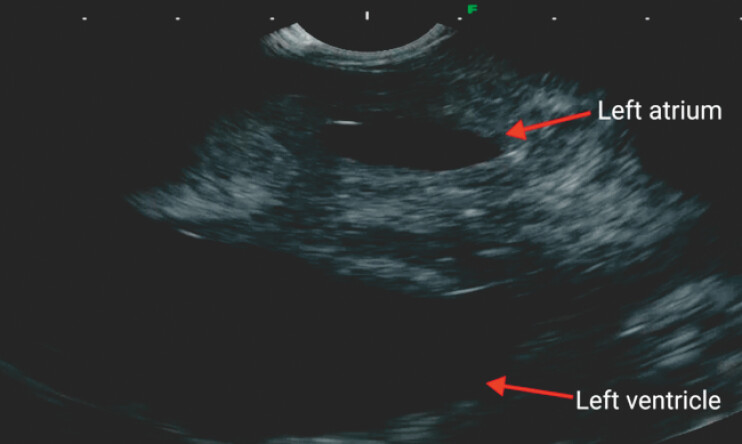
EUS demonstrated the left atrium and left ventricle, with the significant thickening of the left atrial wall. EUS, endoscopic ultrasound.

**Fig. 3:**
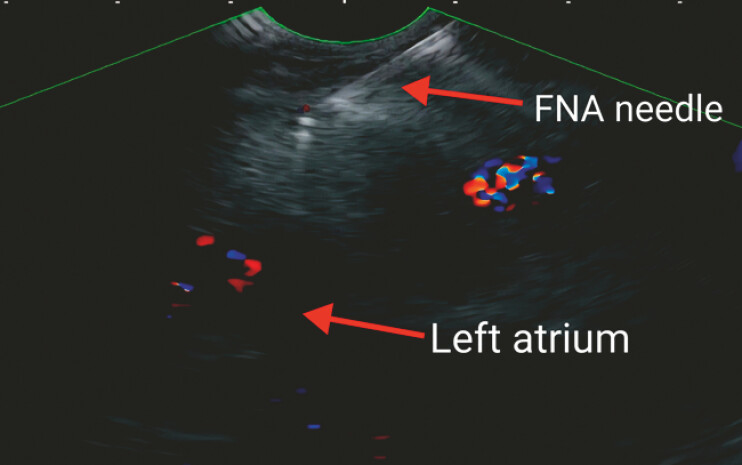
Puncture of the left atrium was performed under EUS guidance. EUS, endoscopic ultrasound.

**Fig. 4:**
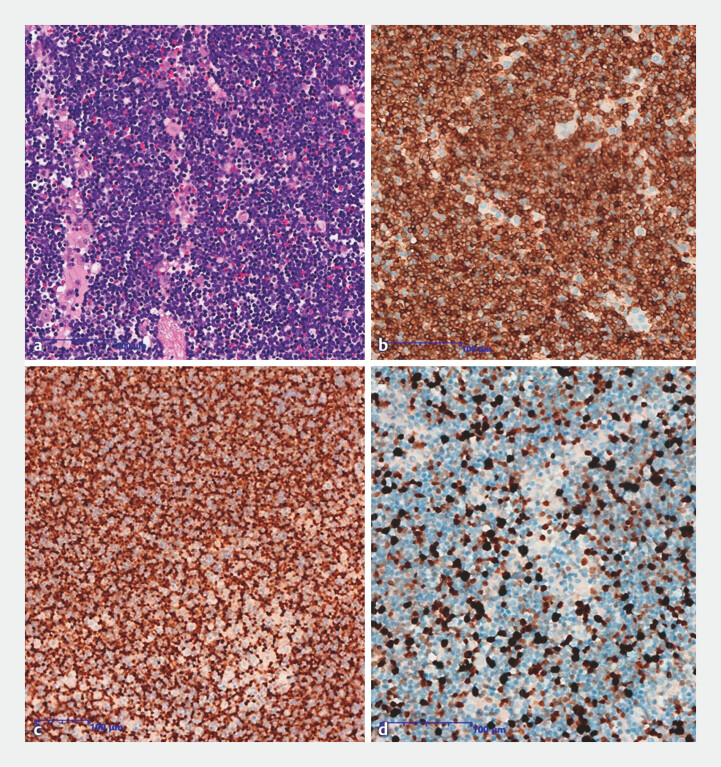
Histopathological and immunohistochemical finding diagnostics of T-lymphoblastic leukemia/lymphoma. **a** HE staining; **b** CD3 immunostaining (positive); **c** TdT immunostaining (positive); **d** Ki-67 immunostaining (positive, approximayely 50% in hotspot areas).

**Fig. 5:**
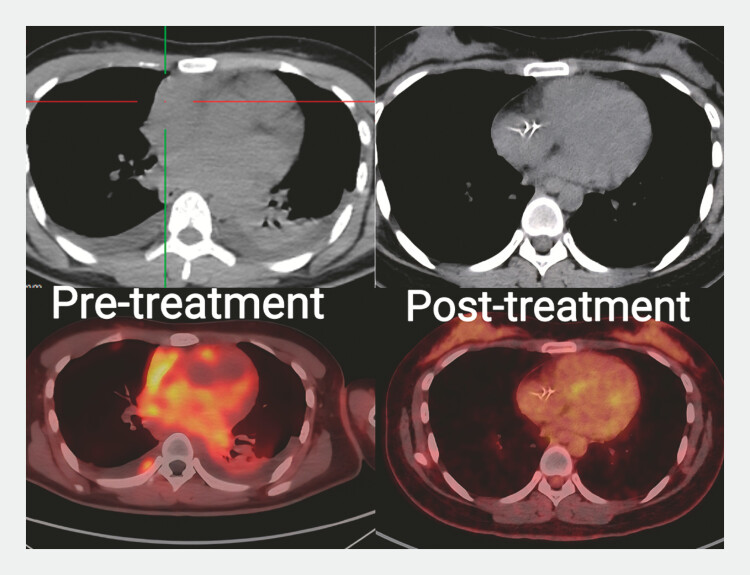
Five months after chemotherapy, the follow-up PET/CT showed significant improvement. PET/CT, positron emission tomography/computed tomography.

A 20-year-old woman was admitted due to progressive chest tightness. Positron emission tomography/computed tomography (PET/CT) showed diffuse metabolic hyperactivity in the heart and local mediastinum, which was considered suspicious for malignancy ( Fig. 1 ). To obtain a definitive pathological diagnosis, transesophageal endoscopic ultrasound-guided fine-needle aspiration (EUS-FNA) was planned. Examination of the mediastinum using a linear 7.5 MHz echoendoscope revealed the significant thickening of the left atrial wall ( Fig. 2 ). Subsequently, the left atrium was punctured under EUS guidance using a 19G needle ( Fig. 3 , Video 1 ). No postoperative complications were observed. EUS-FNA pathological examination with HE staining confirmed lymphoma, with the following immunophenotypic markers ( Fig. 4 ): CD3 (positive), TdT (positive), and Ki-67 (positive, approximately 50% in hotspot areas). A final diagnosis of T-lymphoblastic leukemia/lymphoma(T-LBL/L) was established, providing a clear direction for subsequent treatments. Five months after chemotherapy, follow-up PET/CT showed significant improvement ( Fig. 5 ). The patient remains under an ongoing follow-up.

Puncture of the left atrium was performed under EUS guidance. EUS, endoscopic ultrasound.Video 1

T-LBL/L is a highly aggressive malignancy derived from immature precursor T-cells, characterized by extensive infiltration of immature T-cells into the peripheral blood, bone marrow, and/or extramedullary organs, with the frequent involvement of the central nervous system 1 2 . However, myocardial infiltration is extremely rare. This case demonstrates that EUS-FNA can successfully obtain tissue samples from left atrial lesions, leading to a definitive diagnosis of T-LBL/L and providing critical guidance for clinical management and prognosis. Therefore, the potential value of EUS in non-gastrointestinal pathologies should not be overlooked 3 . Although EUS is currently less commonly applied in non-digestive fields, it still holds significant clinical value. Such cross-disciplinary exploration and application can deepen our understanding of EUS and offer clinicians additional diagnostic and therapeutic insights.

Endoscopy_UCTN_Code_TTT_1AS_2AC
